# Submental liposuction with VASER complicated with hidradenitis suppurativa in neck area: a case report

**DOI:** 10.1093/jscr/rjad318

**Published:** 2023-06-28

**Authors:** Abdullah M Alhusain, Abdulaziz S Almosa, Muhannad Q Alqirnas, Sami I Alissa

**Affiliations:** Plastic and Reconstructive Surgery Division, Surgery Department, Ministry of National Guards Health Affairs, King Abdullah Children’s Specialist Hospital, Riyadh, Saudi Arabia; College of Medicine, King Saud bin Abdulaziz University for Health Sciences, Riyadh, Saudi Arabia; College of Medicine, King Saud bin Abdulaziz University for Health Sciences, Riyadh, Saudi Arabia; Plastic and Reconstructive Surgery Division, Surgery Department, Security Forces hospital, Riyadh, Saudi Arabia

**Keywords:** Hidradenitis Suppurativa, HS, Atypical location, Liposuction, Submental

## Abstract

Hidradenitis suppurativa (HS) is a chronic inflammatory disorder that is characterized by deep-seated painful nodules, classically in the intertriginous skin and apocrine gland-rich areas of the body such as the anogenital, axillary, inframammary and inguinal regions. This is a case of a 35-year-old female, who is known to have gluteal HS, she underwent neck liposuction procedure that was then complicated by anterior neck HS, which is considered as an atypical location. The patient received medical treatment with antibiotics and showed huge improvement. In addition, in patients who do not show response to medical therapy, surgical treatment is usually carried out by incising the area affected and leaving the wound open to be healed by secondary intention or covering it with a skin graft if the area is extensive.

## INTRODUCTION

Hidradenitis suppurativa (HS) is a chronic, recurrent and debilitating follicular-based skin disease [1]. Typical locations of HS include the anogenital, axillary, inframammary and inguinal regions [2]. The disease is insidious; onset is typically postpubertal in otherwise medically free patients, with estimated prevalence of 1% in most contraries [3]. Patients with HS classically present with discomfort and pruritis, followed by tender and firm subcutaneous erythematous nodules, with sizes ranging between 0.5 and 1.5 cm [4]. The primary event of HS is believed to be occlusion of hair follicles, followed by follicular rupture and subsequent dermal inflammation [5]. Moreover, these ruptured follicles can go through reepithelization, which results in the formation of sinus tracts where bacteria and foreign bodies can be housed [6]. Collections can release pus exudate and foul-smelling discharge. Generally, HS can be classified into acute and chronic forms. Hallmarks of the chronic form of HS are abscesses, malodorous exudate drainage from sinuses, overlapping networks of sinuses, dermal inflammation and scars. Treatment modalities of HS include lifestyle modification, medical therapy and surgical treatment. Medical management includes both topical and systemic therapy. Systematic therapy includes antibiotics, anti-inflammatory and hormonal medications. Antibiotic options can include monotherapy with tetracyclines or combination therapy with clindamycin and rifampin [7]. Surgical management is utilized to treat fistulas, nodules and extensive scar tissues in patients with intractable diseases that do not improve with pharmacological management [8].

The location of HS can be found in extremely rare spots on the skin and can occur in any location of the body. Gutierrez *et al*. [9] reported an ectopic HS that is located in a 59-year-old male’s right posterior thigh, whereas Rondags *et al*. reported it in a 28-year-old male’s right dorsal foot [10]. A study published by Boer and his colleagues reported two cases of HS that are located in the waistband of the abdomen. Not only the location can vary and be challenging, but also the disease development. Agiasofitou *et al*. reported a case of a 34-year-old female that had HS since she was 18 and developed new nodules and fistulas on her cesarean section scar; she underwent 5 months ago. The nodules and fistulas appeared after her second cesarean section for 2–3 weeks [12] (Table 1).

**Table 1:** Ectopic hidradenitis suppurativa case reports.

Author	Age (years)	Sex	Location of HS	Comorbidities	Size (cm)
Gutierrez *et al*.	59	Male	Posterior right thigh	Thyroid cancer, HTN	4 × 3.5
Rondags *et al.*	28	Male	Right dorsal foot	Acne conglobate	1 × 2
Boer *et al.*	20	Female	Abdomen (frictional area of her waistband	Morbid obesity, smoker, hypothyroidism	N/A
Boer *et al.*	26	Female	Abdomen (waistband)	Obesity, smoker, hyperhomocysteinaemia	N/A
Agiasofitou *et al.*	34	Female	Abdomen, bilateral axilla	Obesity, smoker	N/A
Boer *et al*. [11]	33	Female	Abdomen, buttock	Obesity	N/A

Liposuction, also known as suction-assisted lipectomy, is a cosmetic procedure that aims to remove access fatty tissue from the subcutaneous space of various body parts to achieve a more desirable body contour [13, 14]. Historically, liposuction was first introduced by Aprad Fischer and Giorgio Fischer [15]. Liposuction is one of the most common esthetic surgical treatments performed globally. In addition, the neck is considered one of the most common areas where liposuction is done.

## CASE REPORT

A 35-year-old female, nonsmoker, known to have psoriasis over the knees, not on any medications, presented complaining of increased weight and skin redundancy over the neck area and underwent VASER liposuction procedure in the submental area in January 2020.

In October 2020, the patient presented to the clinic complaining of painful lesions over her anterior neck area, she reported that the lesions were first noticed 8 months prior to her visit, 1 month post the neck liposuction procedure. The lesions were progressing in size and number, and were associated with pruritis, erythema and on-and-off whitish discharge disrupting her sleep. Upon examination, she had a few tender erythematous inflammatory nodules over her anterior neck. In addition, the patient reported that she tried using different types of topical medications, as a 3-month course of 30 mg isotretinoin was followed but with mild improvement only. She also tried a 1-month course of 100 mg doxycycline OD with no improvement. The patient also reported having similar lesions over her buttocks for 15 years. In that visit, a neck swap from pustules was taken for culture, and a proline 3.0 suture was used to stitch areas over the neck.

**Figure 1:**
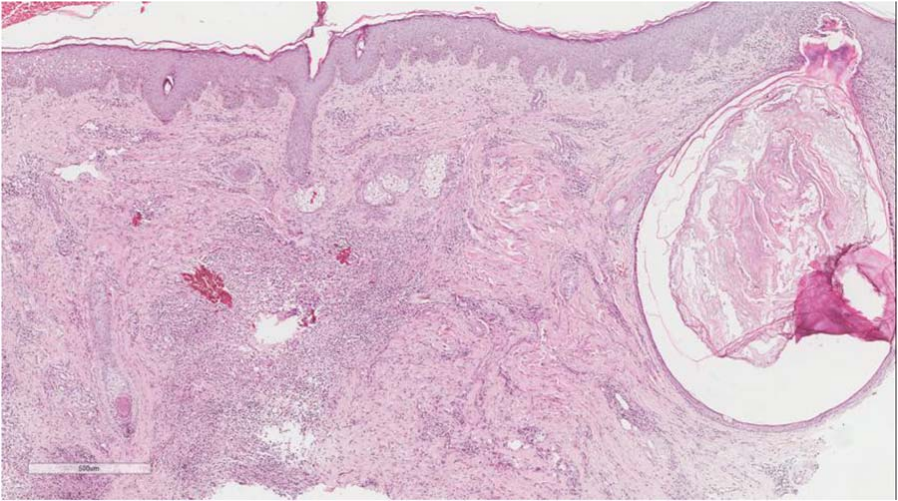
Dilated hair follicle with perifollicular inflammation and adjacent foreign body giant cell reaction secondary to ruptured dilated hair follicle in a background of dermal fibrosis.

**Figure 2:**
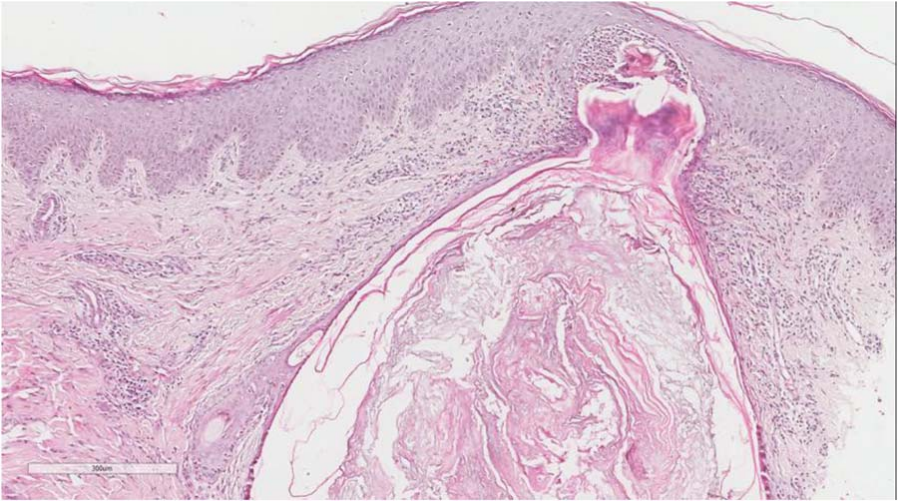
Perifollicular and intrafollicular neutrophilic infiltrate.

**Figure 3:**
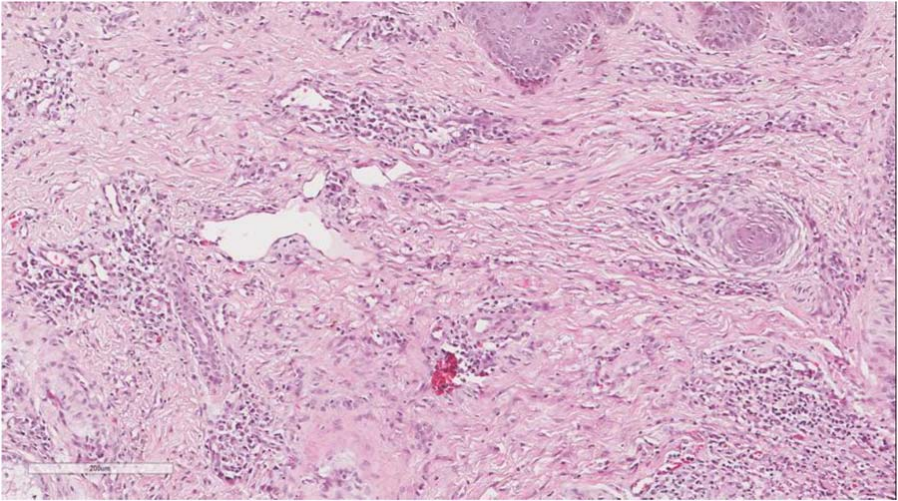
Perivascular lymphoplasmacytic infiltrate that is a common finding in HS.

A 5 mm skin punch biopsy was also taken from the neck. Histopathology showed cystically dilated hair follicle with surrounding fibrosis and heavy inflammatory infiltrate and granulation tissue (Fig. 1), the inflammatory infiltrate is composed of lymphocytes, plasma cells, neutrophils and histiocytes (Fig. 2). The overall morphologic picture was suggestive of follicular occlusion syndrome (Fig. 3). A diagnosis of HS was made, and the patient was started on rifampin 600 mg OD and clindamycin 300 mg BID. A lipid panel was ordered as well and showed a triglyceride level of 1.99 mmol/L (0.7–1.7 mmol/L).

After 1 month, in November 2020, the lesions started to regress after antibiotics initiation, and the patient reported sleep quality improvement as well. Examination revealed multiple erythematous nodules and excoriation over the neck. Her lipid profile showed cholesterol levels of 5.26 mmol/L (3.6–5.2 mmol/L), HDL was 1.38 mmol/L (1–2 mmol/L), LDL was 3.20 mmol/l (1.3–3.4 mmol/L), triglyceride was 1.49 mmol/L (0.7/1.7 mmol/L). Thyroid hormone panel showed T4 level of 10.2 ug/dL (4.6–12 ug/dL), with TSH level of 2.267 mU/L (0.4–5 mU/L). ANA was 1:80 (>1:160 is positive). The fasting glucose level was 4.46 mmol/L (3.9–5.5 mmol/L). The patient was started on chlorohexidine, rifampin 300 mg BID and clindamycin 300 mg BID.

After 2 months, in January 2021, the patient was seen and she reported improvement as lesion sizes were controlled, there was no pain, no pruritis, no discharge and no new lesions. The plan was to continue systemic antibiotics, and topical clindamycin was prescribed. She had another follow-up 1 month later, in February 2021. The patient reported huge improvement, with no new lesions. She then received 5 grams of intralesional Kenalog injection. Systemic antibiotics were stopped, and the patient was instructed to continue topical clindamycin BID for 30 days.

## DISCUSSION

HS is an inflammatory disorder characterized by chronic deep-seated painful nodules, classically in the intertriginous skin and apocrine gland-rich areas of the body. Typical locations of HS include the anogenital, axillary, inframammary and inguinal regions. Patients with HS classically present with discomfort and pruritis, followed by tender and firm subcutaneous erythematous nodules. HS can be classified into acute and chronic forms. HS manifests acutely as a few deep-seated nodules. Hallmarks of the chronic form of HS are abscesses, malodorous exudate drainage from sinuses, overlapping networks of sinuses, dermal inflammation, scars of both atrophic and hypertrophic types.

## CONFLICT OF INTEREST STATEMENT

None declared.

## FUNDING

None.

## DATA AVAILABILITY

The authors confirm that the data supporting the findings of this study are available within the article and its supplementary materials.
